# Classification of multiple primary lung cancer in patients with multifocal lung cancer: assessment of a machine learning approach using multidimensional genomic data

**DOI:** 10.3389/fonc.2024.1388575

**Published:** 2024-05-03

**Authors:** Guotian Pei, Kunkun Sun, Yingshun Yang, Shuai Wang, Mingwei Li, Xiaoxue Ma, Huina Wang, Libin Chen, Jiayue Qin, Shanbo Cao, Jun Liu, Yuqing Huang

**Affiliations:** ^1^ Department of Thoracic Surgery, Beijing Haidian Hospital (Haidian Section of Peking University Third Hospital), Beijing, China; ^2^ Department of Pathology, Peking University People’s Hospital, Beijing, China; ^3^ Department of Medical Affairs, Acornmed Biotechnology Co., Ltd, Beijing, China

**Keywords:** MPLC: multiple primary lung cancer, IM: intrapulmonary metastasis, NSCLC: non-small cell lung cancer, GGO: ground-glass opacity, comprehensive genomic characteristics, molecular classifier, machine learning

## Abstract

**Background:**

Multiple primary lung cancer (MPLC) is an increasingly well-known clinical phenomenon. However, its molecular characterizations are poorly understood, and still lacks of effective method to distinguish it from intrapulmonary metastasis (IM). Herein, we propose an identification model based on molecular multidimensional analysis in order to accurately optimize treatment.

**Methods:**

A total of 112 Chinese lung cancers harboring at least two tumors (n = 270) were enrolled. We retrospectively selected 74 patients with 121 tumor pairs and randomly divided the tumor pairs into a training cohort and a test cohort in a 7:3 ratio. A novel model was established in training cohort, optimized for MPLC identification using comprehensive genomic profiling analyzed by a broad panel with 808 cancer-related genes, and evaluated in the test cohort and a prospective validation cohort of 38 patients with 112 tumors.

**Results:**

We found differences in molecular characterizations between the two diseases and rigorously selected the characterizations to build an identification model. We evaluated the performance of the classifier using the test cohort data and observed an 89.5% percent agreement (PA) for MPLC and a 100.0% percent agreement for IM. The model showed an excellent area under the curve (AUC) of 0.947 and a 91.3% overall accuracy. Similarly, the assay achieved a considerable performance in the independent validation set with an AUC of 0.938 and an MPLC predictive value of 100%. More importantly, the MPLC predictive value of the classification achieved 100% in both the test set and validation cohort. Compared to our previous mutation-based method, the classifier showed better κ consistencies with clinical classification among all 112 patients (0.84 *vs*. 0.65, *p* <.01).

**Conclusion:**

These data provide novel evidence of MPLC-specific genomic characteristics and demonstrate that our one-step molecular classifier can accurately classify multifocal lung tumors as MPLC or IM, which suggested that broad panel NGS may be a useful tool for assisting with differential diagnoses.

## 1 Introduction

Lung cancer is one of the most commonly diagnosed cancer and the leading cause of cancer-related death worldwide (1, 2). Recently, multifocal lung cancer has been detected more frequently, which may be attributed to the advancement of imaging diagnostic technology and the emphasis on early lung cancer screening (3), and the identification of multifocal lung cancer has become an increasingly common clinical problem. Nevertheless, no accurate method was built to distinguish multiple primary lung cancer (MPLC) from intrapulmonary metastases (IM), which is extremely important for the clinical management of lung cancer patients since it affects staging, prognostication and therapeutic choices (4). Indeed, MPLC tends to confer lower staging and a better prognosis than IM, with the main therapies being radical surgery and stereotactic body radiotherapy (5), while IM may need aggressive chemotherapy or targeted therapies (4, 6–10).

In 1975, Martini and Melamed first proposed criteria to distinguish MPLC and IM. Their criteria, based on pathological features, have been widely put into routine clinical use, but it is still challenging to separate IM from MPLC when histological types are identical in the absence of molecular characteristics. With the development of molecular biology and next-generation sequencing (NGS), researchers have been exploring the use of genomic technologies for the classification of lung cancers, but most studies only focus on one or a few genes for classification purposes (3, 11–16) while molecular profiling of MPLC rarely reported (17). Despite the clinical utility in defining tumor lineages, driver mutations have been reported to lead to misclassification of tumor lineages in some challenging cases (18). Currently, several studies observed 27% - 33% discordance with the histological or clinicopathologic criteria by a few hot spot genes (18–20) and no studies have used machine models based on comprehensive genomic characteristics to identify these two diseases.

More comprehensive molecular characteristics may have important implications for our understanding of tumor biology and differential diagnosis in MPLC. Intratumor heterogeneity (ITH) may be required for tumor evolution and has been detected with respect to genetic alterations, which originate and accumulate clonally or subclonally in the course of tumor progression and response to therapy (21–23). Genomic instability makes cancer cells particularly prone to accumulate genetic alterations and has been shown to increase in human metastases (24).

Here, we comprehensively examined the genomic profiles of MPLC and developed a novel random forest (RF) model by using molecular features that are significantly different between MPLC and IM to separate them. To the best of our knowledge, this is the first model by machine learning to identify MPLC using integrated multidimensional molecular features.

## 2 Materials and methods

### 2.1 Study design and participants

**Figure 1:**
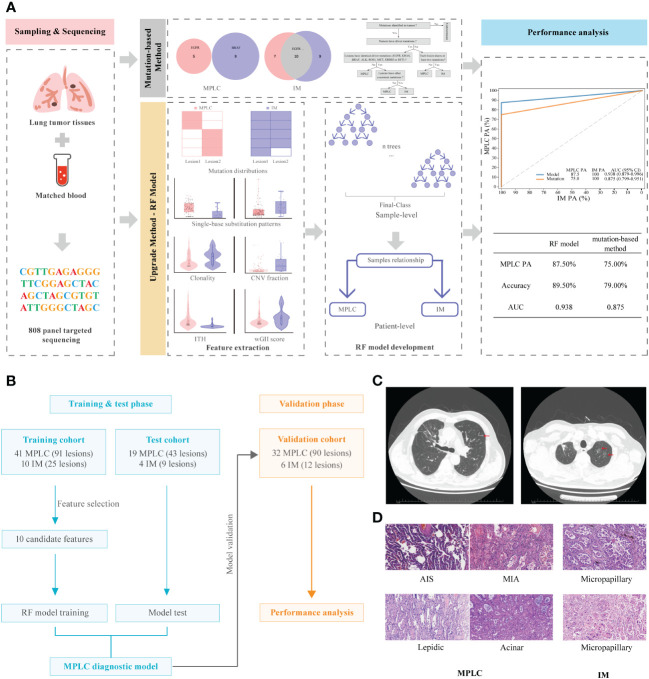
Graphical summary of the study design and participant flow diagram. **(A)** Schematic overview of the study design. **(B)** Participant flow diagram for algorithm development and clinical validation. **(C)** Lung computed tomography (CT) scan. Red arrows indicate sites of lesions. **(D)** Histologic features of lesions of MPLC and IM. Hematoxylin-eosin staining (200×) shows main histopathological images, including AIS, MIA and IAC subtypes (lepidic, acinar and micropapillary). MPLC, multiple primary lung cancer; IM, intrapulmonary metastasis; AIS, adenocarcinoma in situ; MIA, minimally invasive adenocarcinoma; IAC, invasive adenocarcinoma.

The overall study design is illustrated in **Figures 1A, B**. We expanded the study population and upgraded the diagnostic approach from our previous study (11). The main inclusion criteria for patients used to develop and validate the diagnostic classifiers included patients with complete clinicopathological information, a confirmed diagnosis of lung cancer, and tissue samples available for NGS who had at least two lung cancer lesions. The main exclusion criteria included patients with inconclusive histologic decisions for MPLC or IM based on histologic analyses, those with suspected lung metastasis of cancers other than lung cancer and those who received neoadjuvant therapies. A total of 112 Chinese lung cancer patients who underwent surgical resection or biopsy by endobronchial ultrasound-guided transbronchial needle aspiration (EBUS-TBNA) at the Department of Thoracic Surgery of Beijing Haidian Hospital between November 2018 and July 2021 were enrolled. We separated 121 tumor pairs from 74 patients retrospectively into training and test cohorts in a ratio of 7:3 to build the model. In the training phase, we analyzed the molecular feature of 41 MPLC (91 lesions) and 10 IM patients (25 lesions) to find variant candidate biomarkers. In the test phase, 19 patients who were diagnosed with MPLC (43 lesions) and 4 patients with IM (9 lesions) were retrospectively recruited to test the performance of the model. In the validation phase, we prospectively recruited 38 patients between August 2021 and May 2022 as an independent validation cohort to verify the model, including 32 patients with MPLC and 6 patients with IM, consisting of 90 lesions and 12 lesions, respectively.

Two pulmonary pathologists blinded to clinical and genomic data performed independent histologic reviews. The multifocal lung cancers in each patient were diagnosed as MPLC or IM based on American College of Chest Physicians (ACCP) guidelines (25). According to the ACCP criteria, tumors with the same histological subtype located in different lung lobes without lymph node metastasis or systemic metastasis, and the time period between tumors in a pair was less than 4 years were considered as MPLC. IM was associated with lymphatic or systemic metastases and/or an interval of less than 2 years. In addition, different histological subtypes, tumors with the same histological subtype but different mutations, and carcinoma *in situ* were defined as MPLC. The main radiological and pathological features of the tumors in these patients are shown in **Figures 1C, D**, respectively.

### 2.2 Sample preparation and targeted multigene panel sequencing

DNA was extracted from tissues and matched blood. White blood cell samples and fresh tissue samples were extracted using a Blood/Cell/Tissue genomic DNA extraction kit (TIANGEN). Formalin-fixed, paraffinembedded (FFPE) tissue samples were extracted using the GeneRead DNA FFPE Kit (Qiagen). All extractions were performed in accordance with the manufacturer’s instructions. Targeted sequencing was performed using an AcornMed panel, which targeted 808 cancer-related hotspot genes. Target‐enriched libraries were pooled and then sequenced on the NovaSeq6000 System (Illumina Inc.) with 150 bp paired-end sequencing.

### 2.3 Sequence alignment and variant annotation

The raw sequencing reads were first subjected to quality control by trimming adaptor sequences and removing the reads with poly-N and low quality preprocessed by FASTP (26). Then, high-quality reads were aligned to a human reference genome (GRCh37) with Burrows-Wheeler Aligner (BWA) (27), and duplicate reads by PCR were removed by Picard tools. The subsequent data preprocessing and variant calling were based on the Sentieon Genomics pipeline (28). Single-nucleotide variants (SNVs) and small insertions or deletions (INDELs) were analyzed using Sentieon Genomics. Matched genomic DNA from white blood cells was used as a control. The recommended parameters were used, including 1) a mutation allele frequency (AF) at least 1% for tumor tissue DNA; 2) ignoring all silent mutations; 3) at least 10 high-quality supporting reads. SNVs and INDELs were annotated with ANNOVAR (29). Somatic copy number analysis was performed using PureCN (30). GISTIC 2.0 (31) software was used to identify significant aberrations of broad and focal events and to estimate the arm-level copy number status based on the segmented copy number profiles of PureCN. The driver gene hotspot mutations were defined as described previously (11).

### 2.4 wGII, clonal status and ITH estimation

The wGII (weighted genomic integrity index), clonal status and ITH analyses were performed based on ABSOLUTE (32). wGII was determined by the total length of gain plus the loss region divided by chromosome size. Clonal mutation was detected according to the value of the cancer cell fraction (CCF), which was the fraction of tumor cells carrying this mutation within a sequencing sample (32). Mutation was classified as clonal if the estimated CCF was > 0.9 and the Pr (clonal) was > 0.5 and as subclonal otherwise (33). ITH was defined as the ratio of the sum of the subclonal SNV and CNV numbers to the sum of clonal SNV and CNV numbers.

### 2.5 Analysis of mutational signatures and single-base substitution patterns

The mutational signatures with 96 mutation types were extracted for the MPLC and IM groups using the R package MutationalPatterns (34). This algorithm, termed NMF (35), was used to solve the well-known blind source separation problem, which separated the original signal from a set of mixed signals. After that, we calculated the cosine similarity value between these signatures and COSMIC mutational signatures (V2). Single-base substitution pattern (transition or transversion) analysis was performed with the R package maftools (36).

### 2.6 Machine learning algorithm for model development

The MPLC classifier was established using the random forest algorithm, a machine learning dimension reduction strategy based on the construction of thousands of classification or regression trees. We selected characteristics of significant differences between MPLC and IM as candidate features to build the model. The training datasets were used for a grid search of the best parameters and determining the best threshold value with the maximum Youden index of the RF model by 10 k-fold cross-validation. We chose the model that maximized the area under the curve (AUC) during cross-validation. The test datasets were used for assessing the performance of the model, and an independent validation set was used to further validate the classifier. To minimize overfitting, a single patient was maintained as the smallest unit when defining the training and test sets, and all samples belonging to the same patient were considered together as a group in the training and test sets. We reported performance as the AUC and assessed percent agreement (PA) for MPLC and IM PA at a specified score threshold.

### 2.7 Statistical analysis

Statistical analysis was performed with GraphPad Prism 8.0 software. The Wilcoxon rank-sum test, chi-square test and Fisher test were performed when the rate or percentage was compared for significance. Mutation spectrum figures were made with R software. Differences in continuous variables between the groups were analyzed by the Mann-Whitney U test or one-way ANOVA. Pearson correlation coefficients were calculated to evaluate the relatedness of mutations between each pair of samples. The consistency of different classification methods was assessed using the kappa test with SPSS version 23.0. The AUC of the receiver operating characteristic (ROC) curve with a 95% confidence interval (CI) was calculated using the R package pROC (37). The random forest analyses were performed using the Python package SKLearn. A two-sided *p* <.05 was considered statistically significant.

## 3 Results

### 3.1 Characteristics of patients with MPLC and IM

The clinical characteristics of 270 tumors from 112 patients with multifocal lung cancers included in the study are summarized in **Table 1**. The three cohorts had similar clinical characteristics, including age, sex and smoking history (all *P* > 0.05). Among the patients, 88 had two tumors, and 24 had more than two tumors, including 13 patients with 3 tumors and 11 patients with more than 3 tumors (range, 4 - 8).

**Table 1:** Clinical characteristics of patients and tumors with MPLC and IM.

	Training cohort [N = 51]		Test cohort [N = 23]		Independent validation cohort [N = 38]	
Patient characteristics	MPLC [n = 41]	IM [n = 10]	MPLC [n = 19]	IM [n = 4]	MPLC [n = 32]	IM [n = 6]
Age, y (range)	57 (34-76)	58 (31-72)	61 (44-78)	57.5 (41-75)	61 (36-79)	56.5 (36-82)
Gender						
Male	16 (39.0%)	5 (50.0%)	8 (42.1%)	3 (75.0%)	8 (25.0%)	1 (16.7%)
Female	25 (61.0%)	5 (50.0%)	11 (57.9%)	1 (25.0%)	24 (75.0%)	5 (83.3%)
Smoking history						
Smokers	12 (29.3%)	4 (40.0%)	6 (31.6%)	1 (25.0%)	6 (18.8%)	1 (16.7%)
Nonsmokers	29 (70.7%)	6 (60.0%)	13 (68.4%)	3 (75.0%)	26 (81.2%)	5 (83.3%)
Patients with different tumor chronology						
Synchronous	37 (90.2%)	8 (80.0%)	15 (78.9%)	3 (75.0%)	27 (84.4%)	6 (100.0%)
Metachronous	4 (9.8%)	2 (20.0%)	4 (21.1%)	1 (25.0%)	5 (15.6%)	0 (0.0%)
Number of lesions						
2	34 (82.9%)	7 (70.0%)	16 (84.2%)	3 (75.0%)	22 (68.8%)	6 (100.0%)
3	5 (12.2%)	2 (20.0%)	1 (5.3%)	1 (25.0%)	4 (12.5%)	0 (0.0%)
≥4	2 (4.9%)	1 (10.0%)	2 (10.5%)	0 (0.0%)	6 (18.7%)	0 (0.0%)
Tumor characteristics	MPLC [n = 91]	IM [n = 25]	MPLC [n = 43]	IM [n = 9]	MPLC [n = 90]	IM [n = 12]
Stage						
AAH	2 (2.2%)	0 (0.0%)	2 (4.7%)	0 (0.0%)	6 (6.7%)	0 (0.0%)
0	8 (8.8%)	0 (0.0%)	6 (14.0%)	0 (0.0%)	10 (11.1%)	0 (0.0%)
I	78 (85.7%)	0 (0.0%)	34 (79.1%)	1 (11.5%)	72 (80.0%)	0 (0.0%)
II	2 (2.2%)	0 (0.0%)	1 (2.3%)	0 (0.0%)	1 (1.1%)	0 (0.0%)
III	0 (0.0%)	4 (16.0%)	0 (0.0%)	4 (44.4%)	0 (0.0%)	4 (33.3%)
IV	1 (1.1%)	21 (84.0%)	0 (0.0%)	4 (44.4%)	1 (1.1%)	8 (66.7%)
Histology						
AAH	2 (2.2%)	0 (0.0%)	2 (4.7%)	0 (0.0%)	6 (6.7%)	0 (0.0%)
AIS	9 (9.9%)	0 (0.0%)	6 (14.0%)	0 (0.0%)	10 (11.1%)	0 (0.0%)
MIA	34 (37.4%)	3 (12.0%)	12 (27.9%)	0 (0.0%)	31 (34.4%)	0 (0.0%)
IAC	42 (46.2%)	22 (88.0%)	19 (44.2%)	9 (100.0%)	43 (47.8%)	12 (100%)
SCC	2 (2.2%)	0 (0.0%)	0 (0.0%)	0 (0.0%)	0 (0.0%)	0 (0.0%)
IMA	1 (1.1%)	0 (0.0%)	4 (9.3%)	0 (0.0%)	0 (0.0%)	0 (0.0%)
SCLC	1 (1.1%)	0 (0.0%)	0 (0.0%)	0 (0.0%)	0 (0.0%)	0 (0.0%)
Tumor location						
LLL	10 (11.0%)	2 (8.0%)	8 (18.6%)	1 (11.1%)	17 (18.9%)	0 (0.0%)
LUL	21 (23.1%)	2 (8.0%)	10 (23.3%)	3 (33.3%)	21 (23.3%)	3 (25.0%)
RLL	14 (15.4%)	2 (8.0%)	8 (18.6%)	0 (0.0%)	15 (16.7%)	2 (16.7%)
RML	12 (13.2%)	4 (16.0%)	2 (4.7%)	0 (0.0%)	3 (3.3%)	0 (0.0%)
RUL	34 (37.4%)	8 (32.0%)	15 (34.9%)	0 (0.0%)	34 (37.8%)	1 (8.3%)
Extrapulmonary	0 (0.0%)	7 (28.0%)	0 (0.0%)	5 (55.6%)	0 (0.0%)	6 (50.0%)
	Training cohort [N = 51]		Test cohort [N = 23]		Independent validation cohort [N = 38]	
Tumor characteristics	MPLC [n = 91]	IM [n = 25]	MPLC [n = 43]	IM [n = 9]	MPLC [n = 90]	IM [n = 12]
Lymphatic metastasis						
YES	1 (1.1%)	18 (72.0%)	0 (0.0%)	5 (55.6%)	2 (2.2%)	12 (100%)
NO	90 (98.9%)	7 (28.0%)	43 (100.0%)	4 (44.4%)	88 (97.8%)	0 (0.0%)

AAH, atypical adenocarcinoma hyperplasia; AIS, adenocarcinoma in situ; MIA, minimally invasive adenocarcinoma; IAC, invasive adenocarcinoma; SCC, squamous cell carcinoma; IMA, invasive mucinous adenocarcinoma; SCLC, small cell lung cancer; LLL, left lower lobe; LUL, left upper lobe; RLL, right lower lobe; RML, right middle lobe; RUL, right upper lobe.

### 3.2 The mutational landscape of MPLC and IM

To explore molecular biomarkers to differentiate the two diseases, the mutational alterations of MPLC and IM patients in the training cohort were thoroughly investigated. The mutational spectrum of all lesions is shown in **Supplementary Figure 1**. A total of 71 driver-gene hotspot mutations were detected in 90.24% (37/41) of MPLC patients, and the *EGFR* L858R, *EGFR* 19del and *KRAS* G12 mutations were the most common (**Supplementary Figures 2A, B**). Further analysis revealed that there was a high discordance of driver mutations (77.0%, 47/61) between tumors in the same patient with MPLC. In contrast, 23 driver mutations were detected in 90.0% (9/10) of IM patients, and the concordance rate of driver alterations was 100%. However, no significant differences were identified in the frequencies of driver mutations between MPLC and IM patients (**Supplementary Figure 2C**).

### 3.3 Genomic alteration correlation in MPLC and IM among multiple lesions for each patient

**Figure 2:**
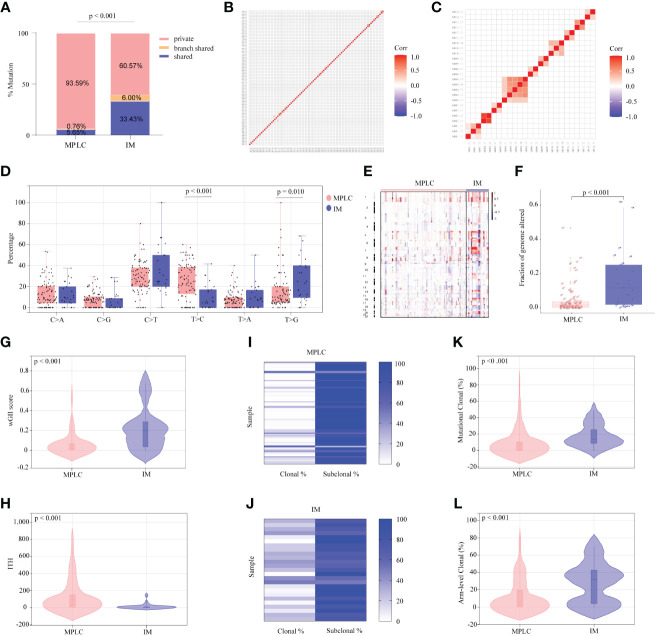
Somatic alterations in MPLC and IM. **(A)** Comparison of the ratio of shared, branch shared, and private mutations for MPLC and IM. Shared mutations are common mutations in all lesions of each patient; branch shared mutations are common mutations in some but not all lesions; and private mutations are unique mutations from a particular lesion. Heatmaps showing the pairwise Pearson correlation coefficients of mutation clusters for MPLC **(B)** and IM patients **(C)**. **(D)** Single-base substitution patterns in MPLC and IM. The box plot shows each type of transition or transversion. The arm-level somatic copy number alteration profiles for samples from MPLC and IM as revealed by GISTIC 2.0 **(E, F)**. **(E)** The heatmap shows the distribution of SCNVs for MPLC and IM samples. Each row represents the copy-number profile of a tumor sample across chromosomes 1 to 22. Red indicates SCNV gain, and blue indicates SCNV loss. **(F)** The boxplot shows the fraction of SCNVs in MPLC and IM. Violin plots exhibiting the comparison of the wGII **(G)** index and ITH **(H)** in MPLC and IM. The heatmap illustrates the clonality of MPLC **(I)** and IM **(J)**. Violin plots exhibiting the mutational **(K)** and arm-level clonal **(L)** proportion of MPLC and IM. ITH, intratumor heterogeneity; wGII, weighted genomic integrity index.

To further investigate genomic alteration patterns of MPLC and IM, mutations were categorized into shared, branch shared and private mutations. The distribution of all three types of mutations in each of the patients showed that 93.59% of mutations in MPLC patients were private mutations, while IM patients had more shared (33.43%) and branch shared (6.00%) mutations, suggesting that patients with MPLC had high level of interfocal heterogeneity than IMs (**Figure 2A**). At the same time, Pearson correlation analysis was performed to delineate the relationship between mutation clusters in MPLC or IM samples. Samples from the same patient were clustered together, and the results showed that there was limited relatedness between lesions in MPLC patients (**Figure 2B**), demonstrating a high discordance of somatic genetic alterations between tumors, and strong clonal relatedness between lesions in IM patients (**Figure 2C**), indicating that more genes were shared.

### 3.4 Mutational signatures of MPLC and IM

The status of single-base nucleotide substitution was not the same between the two groups. A predominance of the T > C transition in MPLC (*P*< 0.001) and a high frequency of the T > G transversion in IM (*P* = 0.010) were identified, with the proportion of other types being nearly the same (**Figure 2D**). Three mutational signatures were identified in MPLC and IM patients (**Supplementary Figures 3A, B**). Signature 5 (exhibited strand bias for T > C substitutions in the ApTpN context) was observed in both MPLC and IM with an unknown cause. There was a very strong enrichment of Signature 1 (associated with age) in MPLC. Three *de novo* mutational signatures were identified in MPLC and IM. These results suggested that the mutational signatures of MPLC were different from that of IM.

### 3.5 Copy number alterations and chromosome instability in MPLC and IM

Analyses of arm-level somatic copy number variations (SCNVs) by GISTIC 2.0 revealed that more amplified and lost segments were detected in IM, with a significantly higher fraction of SCNVs than that in MPLC (median, 0.110498 *vs*. 0.015841, *P* < 0.001) (**Figures 2E, F**). We identified some amplified segments that harbored several known oncogenes, such as *EGFR* (7p11.2), *BRAF* (7q34), *MYC* (8q24.21) and *TERT* (5p15.33) in MPLC, as well as *EGFR* (7p11.2) in IM. We also identified some lost segments, including *EGFR* (7p11.2), *MET* (7q31.2) and *RET* (10q11.21), in MPLC as well as *CDKN2A* (9q21) in IM. We assessed wGII and observed that the majority of tumors showed low-to-moderate genomic instability (median of 0.19884 and 0.0181 per tumor in IM and MPLC, respectively). We found that IM harbored significantly higher wGII scores than MPLC (**Figure 2G**, *P* < 0.001), indicating a higher degree of malignancy in IM.

### 3.6 Intratumor heterogeneity and clonality of somatic mutations in MPLC and IM

Finally, the intratumor heterogeneity and clonal architecture of MPLC and IM were explored. We found the ITH of IM was significantly lower than that of MPLC, with a median of 3.07 and 42 per tumor in IM and MPLC, respectively (**Figure 2H**, median, 42.00 *vs*. 3.07, *P* < 0.001). IM had higher proportion of clonal mutations than MPLC (**Figures 2I, J**, median, 24.56% *vs*. 2.13%, *P* < 0.001), as well as both mutational clonal (**Figure 2K**, median, 13.89% *vs*. 1.30%, *P* < 0.001) and arm-level clonal (**Figure 2L**, median, 32.14% *vs*. 0%, *P* < 0.001) mutations. All of the above results indicated that higher level of ITH and lower proportion of clonal mutations may be characteristic of the primary tumor in early NSCLCs.

### 3.7 Development and validation of the diagnostic classifiers

We constructed a prediction model through the random forest algorithm using ten candidate markers that had significant differences between MPLC and IM, including the number of common mutation sites, common hot driver mutation sites and other common mutation sites per pair, proportion of clonal mutations at the mutational and arm levels, SCNV segment ratio, the fraction of T > C transition and T > G transversion as well as wGII and ITH.

**Figure 3:**
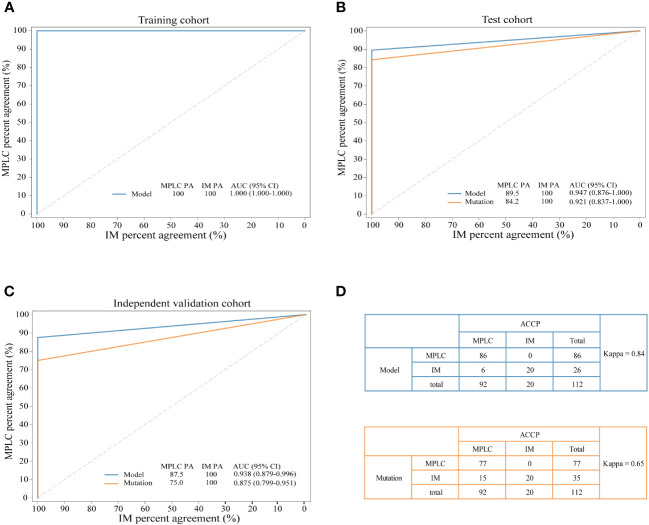
Performance of the novel model and mutation-only classification. Receiver operating characteristic (ROC) curves of the novel diagnostic model and previous molecular method in the training cohort **(A)** test cohort **(B)** and independent validation cohort **(C)** A comparison of the novel diagnostic classifier (upper) and mutation method (lower) for detecting MPLC and IM **(D)** PA, percent agreement; AUC, the areas under the curves.

The combination of two samples from individual patients was used to build the classifier models at the sample level. A total of 121 tumor pairs were assigned in a 7:3 ratio for model training and testing by stratified random sampling. The logical relationship between the sample level and the patient level is that when the clonal relationship of all tumor pairs in the same patient is MPLC, the patient is classified as MPLC; otherwise, it is classified as IM. In the training cohort, the AUC was 1.000 with an optimal threshold at sample level (**Supplementary Figure 4**). We evaluated the model performance at patient level. The classifier successfully classified all MPLC and IM cases in training cohort, yielding a total accuracy of 100% (AUC = 1.000, **Figure 3A**). Then, test datasets were used to verify the RF model. The model separated the two diseases well, with an IM PA of 100% and an MPLC PA of 89.5% (AUC = 0.947, 95% CI 0.876-1.000, **Figure 3B**). Finally, we assessed the performance of the classifiers on an independent validation cohort. The RF model performed equally well, with an IM PA of 100% and an MPLC PA of 87.5% (AUC = 0.938, 95% CI 0.879-0.996, **Figure 3C**).

### 3.8 Comparison of the performance of the RF model with mutation-only classification

Additionally, we compared the performance of the RF model with our previous mutation-only classification (11). For the mutation-based method, the IM PA was 100%, and the MPLC PA was 84.2% (AUC = 0.921, 95% CI 0.837-1.000) in the test cohort (**Figure 3B**). The AUC was 0.875 (95% CI 0.799-0.951), with an IM PA of 100% and an MPLC PA of 75% in the independent validation set (**Figure 3C**). Totally, the RF classifier showed better AUC than our mutation method in both test cohort and validation cohort. Details of the performance of the RF model showed in **Supplementary Table 1**. Among all 112 patients, the RF model showed extremely high agreement with ACCP criteria (κ = 0.84; **Figure 3D**, upper), whereas the κ consistency between our previous mutation-only method and the ACCP criteria was 0.65 (**Figure 3D**, lower). Our results show that the RF classifier has significantly higher consistency with ACCP criteria than the mutation-only method.

**Figure 4:**
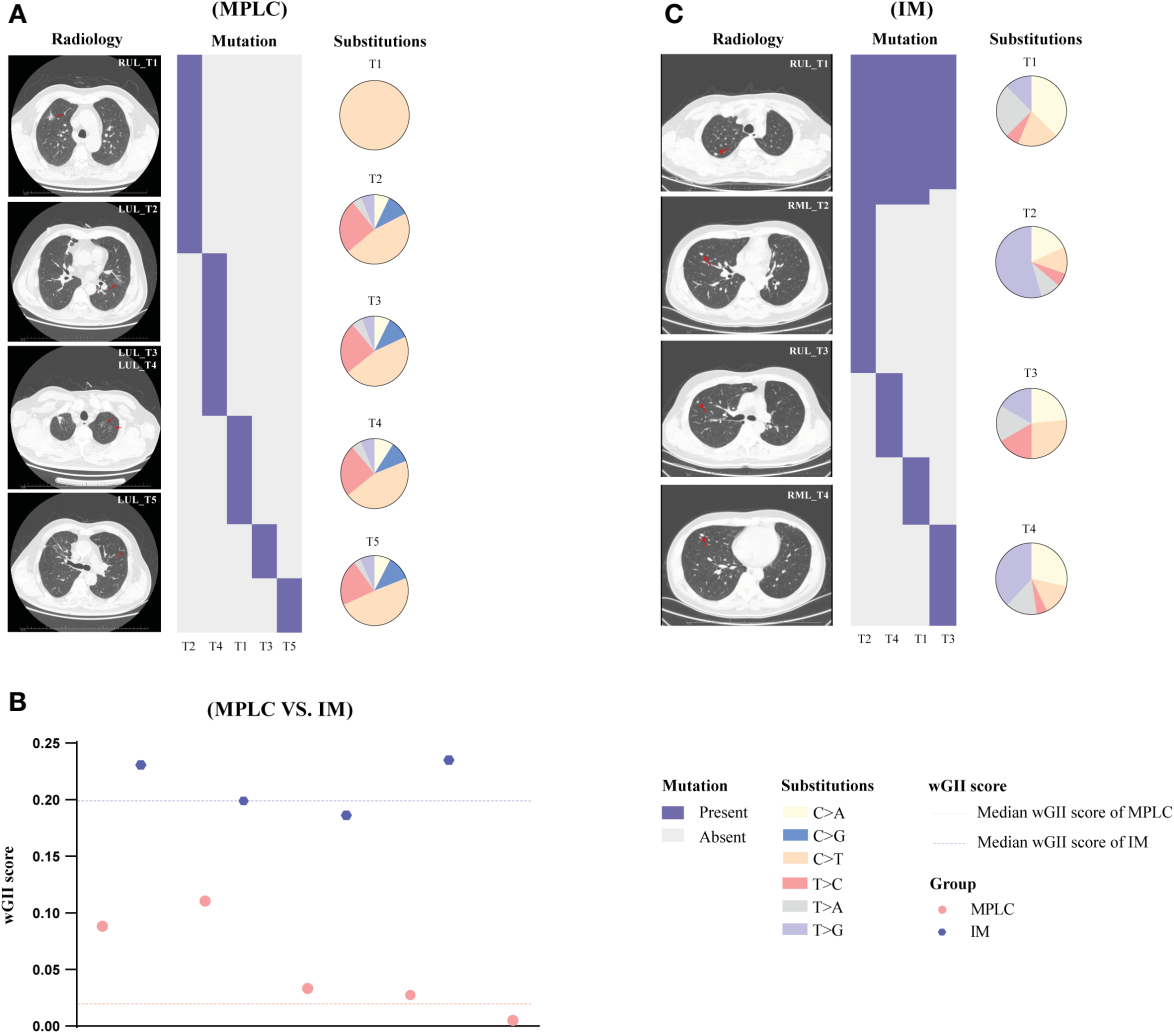
Gene mutation spectrum in case P068 (MPLC) and case M004 (IM). The radiological features and regional distribution of somatic mutations (heatmap) and single nucleotide variations (pie chart) in MPLC **(A)** and IM **(C)**. The wGII score of 9 tumors from two cases **(B)**. Blue and red dashed line represents the median wGII score for patients with IM and MPLC, respectively. LUL, left upper lobe; RUL, right upper lobe; RML, right middle lobe.

Descriptions of two representative cases (P068 and M004) are presented below. We sequenced samples from five tumors of case P068 which clinically diagnosed with MPLC. No shared gene was found in all lesions and different driver hot gene sites were detected in each tumor, including *EGFR* and *BRAF*. All five tumors showed high proportion of C > T (**Figure 4A**) and lower wGII scores (**Figure 4B**). However, in case M004 with IM, eight shared mutation gene sites were detected in four lesions, including *EGFR* and *TP53*. The proportion of T > G (**Figure 4C**) and the wGII scores were high in four lesions (**Figure 4B**). The results of these two patients were clinically consistent with our RF identification model, suggested the feasibility of using multidimensional molecular features to assist clinical diagnosis of multiple lung cancers. **Supplementary Figures 5**, **6** show the regional distribution of all somatic mutations in 270 tumors from 92 MPLC and 20 IM.

## 4 Discussion

In the survey on the management of multiple lung lesions, two-thirds of the responders performed molecular studies to assess the genetic agreements of different lesions. However, the process of tumor metastasis and molecular criteria remain ambiguous (38). We first presented the application of a novel comprehensive molecular classification algorithm for defining MPLC and IM in patients with multiple lung cancers. This finding is encouraging in that one-step molecular diagnosis has a high diagnostic accuracy of 94.6% with an 8% improvement compared to our previous mutation-only method (11), and has a significant improvement over other reported molecular methods with accuracy of about 70% (18–20). The improved diagnostic performance of our RF model showed that larger panels could provide more detailed mutation information of tumors and present far greater promise of genomics in defining tumor lineage.

Our study has several unique features. First, the very high correlation between the classifier algorithm and the expert pathologists’ diagnoses based on ACCP guidelines (25) validates the accuracy of the classifier. Second, the training cohort included a broad range of pathological subtypes, including AAH, AIS, MIA (37.4%), IAC, SCC, IMA, and SCLC, approximating the diversity of stage I MPLCs encountered in clinical practice. In addition, we considered patients who had undergone resections with three or more tumors for the first time. In contrast, most previous studies of genomic profiling compared differences between paired IAC or paired SCC (16, 39, 40). Third, the classifier was trained and tested with a combination of banked and prospectively collected samples to ensure robustness against potential differences in sample handling and collection. Finally, many previous studies were analyses of differential gene mutations alone (3, 13, 16); the investigators did not use these data to build a classification engine. Our approach is a rigorous method for the development of molecular tests that, when properly trained and validated, generalizes well to independent datasets.

A wide range of clinical research about the distinction between MPLCs and IMs has been reviewed in the literature (18, 39). Studies investigated driver mutations, lineage relationships, and somatic rearrangements among tumors by multi-gene large panel NGS (16). By comparison, our one-step molecular diagnostic model offers a more streamlined and standardized approach to analyzing genetic data, reducing the complexity associated with multi-gene panels. This streamlined process enhances efficiency and facilitates easier interpretation of results, leading to quicker and more accurate diagnoses. Additionally, our model utilizes advanced computational algorithms and machine learning techniques, allowing for the identification of complex patterns and relationships within the data that traditional mutation methods may miss. Our approach improves diagnostic accuracy (10-20%), including in the case of multiple lesions. In recent years, radiomics has gained momentum towards the diagnosis of multifocal lesions to provide a patient-based signature (41–43). CT imaging provides valuable anatomical visualization, facilitating the assessment of lesion morphology, size, and distribution. Furthermore, it allows for dynamic monitoring of lesions over time, aiding in the observation of growth patterns. Image AI methods offer rapid and non-invasive analysis of medical imaging data, facilitating quick diagnosis and treatment planning at a lower cost. Nevertheless, CT imaging may have limitations in accurately characterizing lesions, the diagnostic accuracy of CT imaging methods is generally 75-88% (42), which is lower than that of molecular diagnostic techniques. It is also subject to variability in interpretation by individual expertise and involves radiation exposure risks. Image AI interpretation relies on the quality and quantity of training data, and their performance may be affected by variations in imaging protocols or equipment, which is not mature yet.

In this study, we aimed for high IM percent agreement at patient level (> 90%) because of higher clinical utility. Our model can provide an excellent classification performance compared with our previous mutation-only method. In the test and validation cohort, IM PA increased significantly on application of our algorithm. Interestingly, several IMs, in whom part of the tissue was obtained through endobronchial ultrasound-guided transbronchial needle aspiration, have a high concordance between clinical diagnosis and molecular diagnosis by our both molecular methods, which indicates that the diagnosis was not significantly affected by tissue heterogeneity due to the acquisition of samples.

Of the 10 features rigorously selected, common hotspot and driver mutations have been the most thoroughly studied (3, 18). Interestingly, many pairs of independent primary tumors from patients shared identical *EGFR* L858R and 19del and *KRAS* G12C. However, none of the driver events was found to be private in metastases, indicating that the majority of driver diversity accumulated in the primary tumor, which then served as the substrate for the selection of metastasis-competent populations. Consistent with previous reports, canonical cancer gene mutations in *EGFR*, *ERBB2*, and *BRAF* were always truncal as early as the AAH/AIS stage, suggesting that these mutations are very early genomic events before the acquisition of invasiveness (44).

Here, we have provided an analysis of each tumor clonal status, which has shown that heterogeneity and branched evolution are almost universal across MPLC patients. Our study revealed a higher proportion of subclonal mutations (**Figures 2I–L**) and branch mutations (**Figure 2A**) in early-stage MPLC than in advanced-stage IM. We also observed a common pattern of extensive subclonal diversification in MPLC, suggesting a higher level of ITH complexity. In characterizing metastases, we showed evidence of evolutionary bottlenecking, with metastatic lesions being more homogeneous than primary tumors (proportion of clonal variants: 24.56 *vs*. 2.13). This finding suggests that genomic instability processes at the mutational and chromosomal levels are ongoing during tumor development. Moreover, enrolled patients with metastatic lesions had no selection for therapeutic efforts, resulting in fewer subclonal mutations. A pattern of high level of ITH may be characteristic of the primary tumor.

A greater understanding of chromosomal instability is necessary, which can alter the copy number of a multitude of genes simultaneously. We observed widespread wGII for both somatic CNV and mutations in IM patients. In tumors characterized by low ITH and high wGII, metastatic competence is acquired within the most recent common ancestor, which drives rapid dissemination (45). Hence, a low ITH/high wGII pattern may be prevalent in metastatic tumor of patients who are deemed inoperable. Notwithstanding these findings, most features (8 of 10) in the model have rarely been reported to be involved in MPLC and IM. Further investigation of these genomic characteristics might provide insights into the pathogenesis of MPLC at an early stage.

Our study confirmed that most multifocal lesions are tumors of multiple primary synchronous occurrences. We found discordance for 6 patients (P050, P014, P066, P072, P078 and P088) in the RF model, who were diagnosed with MPLC according to the ACCP criteria but were classified as IM according to our final classification. Among them, we identified metastasis can occur among multifocal pure ground-glass opacities (pGGOs) in two cases. This finding suggests that pGGOs can disseminate metastatic lesions, while metastatic lesions can remain pGGOs. Long-term follow-up of these patients will be conducted to further validate the performance of our classifier.

This study had some limitations that warrant future work. First, the number of patients was still limited, especially for IM. Second, lack of independent external central cohort to evaluate the generalizability of the model. Third, survival difference analysis between MPLC and IM in the cohort was not included due to the short follow-up time, but we will follow these patients actively over a long period of time in the future. Despite the recognized limitations of this study, it is becoming apparent that the availability of more comprehensive genomic testing has the potential to be an important addition to the standard staging methods currently used clinically.

In conclusion, a novel diagnostic approach which convenient and promising using comprehensive molecular data can allow differentiation between MPLCs and IMs in a substantial number of cases of multiple lung cancers with multiple pulmonary sites of involvement, which could help doctors with precise decision-making in routine clinical practice.

## Data availability statement

The datasets presented in this study can be found in online repositories. The name of the repository and accession number can be found in the article/Supplementary Material. Accession of the submission is HRA007178 (https://ngdc.cncb.ac.cn/search/specific?db=hra&q=HRA007178).

## Ethics statement

The studies involving humans were approved by Medical Ethics Committee of Beijing Haidian Hospital. The studies were conducted in accordance with the local legislation and institutional requirements. The participants provided their written informed consent to participate in this study. Written informed consent was obtained from the individual(s) for the publication of any potentially identifiable images or data included in this article.

## Author contributions

GP: Conceptualization, Supervision, Data curation, Investigation, Project administration, Formal analysis, Writing – original draft. KS: Conceptualization, Data curation, Formal analysis, Investigation, Writing – original draft, Methodology, Visualization. YY: Conceptualization, Data curation, Formal analysis, Investigation, Writing – original draft. SW: Data curation, Formal analysis, Investigation, Writing – review & editing. ML: Data curation, Investigation, Methodology, Validation, Visualization, Writing – original draft. XM: Data curation, Methodology, Validation, Visualization, Writing – original draft. HW: Methodology, Investigation, Software, Supervision, Writing – review & editing. LC: Methodology, Software, Writing – review & editing, Validation, Visualization. JQ: Methodology, Writing – review & editing, Investigation, Project administration, Supervision. SC: Project administration, Supervision, Writing – review & editing, Resources. JL: Project administration, Resources, Supervision, Writing – review & editing, Investigation. YH: Data curation, Investigation, Writing – review & editing, Conceptualization, Project administration, Resources, Supervision.
