# Supplementary material for: Classification of multiple primary lung cancer in patients with multifocal lung cancer: assessment of a machine learning approach using multidimensional genomic data
Source: Front Oncol. 2024 May 3;14:1388575. doi: 10.3389/fonc.2024.1388575 (PMC11100425; doi:10.3389/fonc.2024.1388575)
Supplement: Supplementary file 1 [file DataSheet_1.docx]

**Additional file 1 for**

**Classification of multiple primary lung cancer in patients with multifocal lung cancer: assessment of a machine learning approach using multidimensional genomic data**

**Figure S1** The mutational landscape between MPLC and IM. Alteration landscape of all lesions from the training cohort, including 41 MPLC **(A)** and 10 IM patients **(B)**. The upper panel shows the numbers of nonsynonymous single‑nucleotide variants, and small insertions or deletions in each tumor. The heat map below shows the top 20 cancer genes with somatic mutations sorted as per the mutation frequency.

**Figure S2** The driver mutations of MPLC and IM. Lollipop plots for amino acid changes resulting from *EGFR* **(A)** and *KRAS* **(B)** mutations with MPLC. Altered loci with high frequency are indicated. Comparison of driver mutational frequencies between MPLC and IM **(C)**.

**Figure S3** Inferred mutational signatures. Three mutational signatures were deciphered from the base substitutions identified in MPLC **(A)** and IM **(B),** respectively.


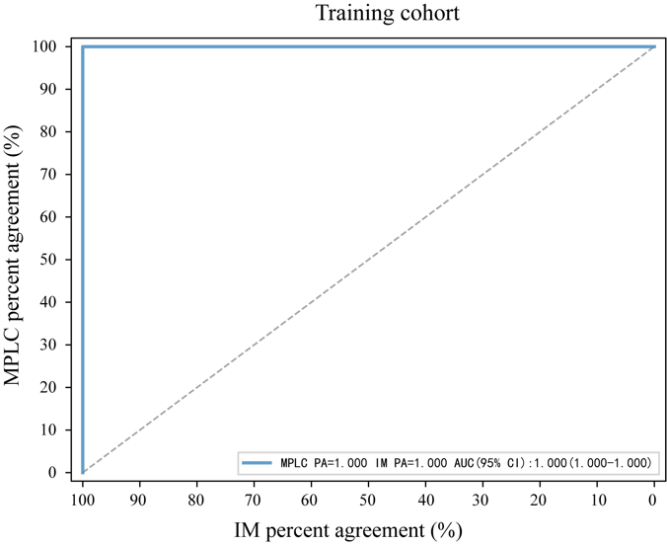


**Figure S4** Receiver operating characteristic (ROC) curves of the novel diagnostic model in training cohort at sample level. PA, percent agreement.

**Figure S5** The mutation distributions of the different tumor lesions in MPLC. Regional distribution of all somatic mutations in multifocal tumors in the same patient.

**Figure S6** The mutation distributions of the different tumor lesions in IM. Regional distribution of all somatic mutations in multifocal tumors in the same patient.
